# Nanoemulsions of Essential Oils: New Tool for Control of Vector-Borne Diseases and In Vitro Effects on Some Parasitic Agents

**DOI:** 10.3390/medicines6020042

**Published:** 2019-03-27

**Authors:** Javier Echeverría, Ricardo Diego Duarte Galhardo de Albuquerque

**Affiliations:** 1 Facultad de Química y Biología, Universidad de Santiago de Chile, Casilla 40, Correo 33, Santiago 9170022, Chile; 2 Laboratório de Tecnologia em Produtos Naturais, Universidade Federal Fluminense, Niterói 24241-002, Brazil

**Keywords:** nanoemulsion, essential oils, vector control, infectious diseases

## Abstract

The control of infectious/parasitic diseases is a continuing challenge for global health, which in turn requires new methods of action and the development of innovative agents to be used in its prevention and/or treatment. In this context, the control of vectors and intermediate hosts of etiological agents is an efficient method in the prevention of human and veterinary diseases. In later stages, it is necessary to have bioactive compounds that act efficiently on the agents that produce the disease. However, several synthetic agents have strong residual effects in humans and other animals and cause environmental toxicity, affecting fauna, flora and unbalancing the local ecosystem. Many studies have reported the dual activity of the essential oils (EOs): (i) control of vectors that are important in the cycle of disease transmission, and (ii) relevant activity against pathogens. In general, EOs have an easier degradation and cause less extension of environmental contamination. However, problems related to solubility and stability lead to the development of efficient vehicles for formulations containing EOs, such as nanoemulsions. Therefore, this systematic review describes several studies performed with nanoemulsions as carriers of EOs that have larvicidal, insecticidal, repellent, acaricidal and antiparasitic activities, and thus can be considered as alternatives in the vector control of infectious and parasitic diseases, as well as in the combat against etiological agents of parasitic origin.

## 1. Introduction

### 1.1. Essential Oils and Biological Activity

In recent years, the use of essential oils (EOs) from aromatic plants as low-risk insecticides, antifungals, antifeedants, antivector growth regulators, oviposition deterrents and repellents [1,2,3,4] has increased due to its popularity among organic farmers and environmentally conscious consumers.

The production of EOs is mainly carried out through procedures of hydrodistillation, steam distillation, dry distillation, or mechanical cold pressing of plants [5]. The most widely used preparation method is based on the Clevenger steam distillation apparatus, which has been adapted and expanded for industrial scale production. Recently, modern EO extraction methods have begun to be used, including microwave-assisted processing and supercritical fluid extraction [6].

A typical EO can contain, on average, between 20 and 80 different compounds. The constituents of EOs mainly belong to two phytochemical groups: terpenoids (monoterpenes and sesquiterpenes of low molecular weight) and, to a lesser extent, phenylpropanoids (volatile phenolic compounds). The terpenoids are the main constituents of the EOs. The monoterpenes present in the EOs may contain terpenes which are hydrocarbons (α-pinene and D-limonene), alcohols (cadinol), aldehydes (cinnamaldehyde and citronellal), ketones (thujone), ethers (1,8-cineol) and lactones (artemisin). The sesquiterpenes have a wide variety of structures, more than 100 skeletons, since the lengthening of the chain to 15 carbons increases the number of possible cyclizations. Aromatic compounds are less common and include phenylpropanoids (*p*-allylanisole) and phenolic compounds (thymol).

The composition of EOs is very diverse in different plant species. An example of this is the eucalyptus (*Eucalyptus globulus*), whose main constituent of the EO is monoterpene 1,8-cineol, while in coriander (*Coriandrum sativum*), the sesquiterpene linalool is the most abundant. In turn, within the same species of plants, the existence of chemotypes is very common. For example, thyme (*Thymus vulgaris*) has numerous chemotypes named after the main compound, for example, thymol, carvacrol, terpineol and linalool.

In botanical terms, EOs are produced in approximately 17,500 species of higher aromatic plants, belonging mainly to a few families, including *Myrtaceae*, *Lauraceae*, *Lamiaceae* and *Asteraceae*. The synthesis and accumulation of EOs in plants are associated with the presence of complex specific and highly specialized secretory structures, such as glandular trichomes (*Lamiaceae*), secretory cavities (*Myrtaceae*, *Rutaceae*) and resin ducts (*Asteraceae*, *Apiaceae*). Depending on the species taxonomically considered, the EOs can be stored in various plant organs, for example, in flowers, leaves, wood, roots, rhizomes, fruits and seeds [7].

At the biological level, the EOs have important effects as repellents, insecticides and growth reducers in a great variety of insects. They have been used effectively to control phytophagous insects pre- and post-harvest, as insect repellents with vectors of diseases and for the control of domestic and/or garden insects. The mechanism of action of the compounds present in EOs on insects is mainly through neurotoxic effects, involving several modes of actions, in particular, through the inhibition of acetylcholinesterase (AChE) [8], functionality disruption of gamma-aminobutyric acid (GABA) receptors [9], and as agonist of octopaminergic system [10].

Although the biological effects of the individual chemical components of the EOs are generally known, the toxicology of their combinations or mixtures is a much more difficult aspect to evaluate. However, one of the most outstanding and attractive characteristics of the EOs is that they are, in general, low-risk products on animals, and their environmental persistence is short. Their toxicity for mammals is low, having values of oral LD_50_ that varies from 1000 to 2000 mg kg^−1^ in rats. In addition, due to their use as medicines, some EOs are relatively well studied experimentally and clinically.

The toxicity of EO components has been divided into three structural classes based on the toxicological potential [11]: Class I (low functionality, low oral toxicity, e.g., limonene), Class II (some functionality, intermediate toxicity, e.g., menthofuran) and Class III (reactive functionality, high potential toxicity, e.g., elemicin). Based on this classification and other toxicological criteria, different procedures have been developed to evaluate the safety of the use of EOs [12].

Moreover, the EOs also could be promising antiprotozoal agents, opening perspectives to the discovery of more effective drugs of vegetal origin for the treatment of related diseases by targeting the etiological agents. In several species with antiprotozoal activity, the compounds camphor, carvacrol, eugenol, terpinen-4-ol, or thymol were the main oil constituents, which denotes a supposed variety in its mechanisms of action, since these substances belong to different structural groups [13,14].

### 1.2. Nanotechnology and Nanoemulsions

Nanotechnology is a transdisciplinary and promising field of science for its diverse potential of application, ranging from the field of electronic and mechanical engineering, telecommunications, civil construction, industrial chemistry, to practical use in the health area, as the development of nanostructured drugs, biological carriers and sensors for diagnosis [15,16]. With the advancement of technologies in electron microscopy in the 1980s, it became possible to observe, study, manipulate and develop nanostructured systems, which led a technological revolution in several fields of science [17].

Nanometric materials have a size in the range of 0.1 to 200 nm and present particular physicochemical properties in relation to structured materials on a macroscopic scale [18]. In the field of biological and health sciences, nanotechnology has provided a promising alternative for research and development of drug delivery systems, as allow better delivery of the drug to its place of action, a factor that provides a series of patient benefits such as increased therapeutic efficacy, dose reduction, lower number of administrations, and a decrease in side effects. Still, regarding technical stability, nanosystems have the advantage of protecting active substances against mechanisms of inactivation and degradation, besides providing the incorporation of substances with very different polarity in relation to the matrix, to promote prolonged release and/or targeting of drug action in a certain tissue. These events occur due to different factors, such as the larger contact surface, which promotes greater absorption, distribution and tissue uptake of the drug, in addition to the physicochemical properties of the coating or matrix polymers, which may provide greater affinity with the target tissue or greater protection against physiological barriers in the organism [15,19,20].

An emulsion is a liquid dispersion of two different immiscible liquids. The macroscopic separation of the two different phases is controlled by the addition of the surfactant. When this emulsion system tends to reach nanometric size from 20 to 200 nm, it is called “nanoemulsion”. Nanoemulsions can be formed through the dispersion of the oil in water or water in oil and allow the placement of different active substances. They have some individual characteristics, such as transparency when viewed by the naked eye, and a bluish reflection may be observed, due to the diffusion of light between the nanoparticles, which is known as the Tyndall effect [21]. The extremely small size of the particles also confers greater resistance to the effects of cremation and sedimentation, because the effect of gravity is smaller on them [22].

The surfactant has a vital role in the formation of these nanoemulsion systems. The interfacial tension generated due to the immiscibility of two different liquid systems is reduced through the use of surfactant. This property of lowering the interfacial tension between the phases tends to make the surfactant an essential part of the nanoemulsion system. This formulation process for the nanoemulsion is carried out through the low or high energy emulsification method. The low energy method includes the phase inversion method, while the high energy method includes ultrasound and high-pressure homogenization. In addition, the system displays low interfacial tension, which facilitates easier dispersion of different substances. This fact is a major advantage when the incorporation of highly lipophilic materials, such as EOs, into aqueous systems is necessary [22,23]. Thus, the nanoemulsions have diverse applications in several fields, such as, for example, pharmaceuticals, cosmetics, agriculture, etc. [24]. In the current scenario, different approaches are being carried out in the development of nanoemulsion with an efficient insect repellent, larvicidal and insecticidal activity using EOs.

### 1.3. Essential Oil Nanoemulsion

In recent years, several scientific works have reported the use of nanoemulsions as suitable carriers of active EOs. The easy preparation, simple composition, higher thermodynamic stability, low cost of production and the possibility of production on an industrial scale provide a substantial advantage to this technology through the biological and pharmacological use of EOs [25,26].

Nanoemulsions of EOs are promising tools for combating vector-borne diseases through population control of transmitting agents. This is possible due to the inherent physicochemical properties of the nanometric emulsion system [27]. The nanometer size of the nanoemulsion improves its specificity and delivery target, resulting in greater effectiveness than bulk pesticides. Furthermore, nanoemulsions pesticides have a lower surfactant concentration than microemulsions, and surfactants are considerably more environmentally friendly and are cost-effective and economically viable. EOs dispersed in different nanoformulations can act through several mechanisms of action, such as the deregulation of the growth hormone that tends to stop the insect shedding, which leads to its mortality, as well as enzymatic inhibition, among others [28,29,30]. Finally, recent studies have also reported the biological activity of nanoemulsified essential oils on etiological agents of parasitic origin, which potentially increases the diversification of the use of these nanoemulsions in the control of infectious/parasitic diseases.

## 2. Biological Activities of Essential Oil Nanoemulsions

### 2.1. Larvicidal Activity

*Culex quinquefasciatus* is a mosquito species acting as cyclic vector for *Wuchereria bancrofti*, a human filarial nematode, that causes filariasis. This disease mainly causes lymphedema, which in turn leads to patient morbidity and is potentially fatal. In 2014, Sugumar et al. [31] developed a eucalyptus EO-based nanoemulsion that showed larvicidal activity against *C. quinquefasciatus*. The treated larvae exhibited lower protein levels, as well as significant reduction in the levels of acetylcholinesterase and acid/basic phosphatase. In this way, the developed nanoemulsions can be used as a safe and effective alternative in the control of vector-borne filariasis.

Ghosh et al. [32] demonstrated the activity of the nanoemulsionated EO from *Ocimum basilicum* L. against third instar larvae from *Aedes aegypti*, one of the main dengue-transmitting mosquitoes. The EO composition was shown to be 88% of methyl chavicol (estragole), a phenylpropanoid with insecticidal property [33]. Thus, in this work, the hundred-fold dilution of nanoemulsion caused larval mortality of 60 and 70% after exposure periods of 60 and 75 min, respectively. Complete loss of larval viability in this concentration was observed after an exposure period of 90 min. However, the ten-fold diluted nanoemulsion induced 100% larval mortality of *A. aegypti* in 15 min [32].

In 2015, Brazilian researchers developed a nanoemulsion with *Rosmarinus officinalis* EO as an active constituent against 4th instar larvae of *A. aegypti.* The mortality ratio was evaluated after 24 h and 48 h of contact with the nanoemulsion at 250 ppm, which induced 80 and 90% mortality, respectively [34]. 1,8-cineol is the main constituent of this EO and showed potent larvicidal activity [35,36]. Previously in 2005, Prajapati et al. [37] demonstrated that the non-emulsified *R. officinalis* EO exhibited a DL_95_ of 408 ppm after 24 h of contact, which, in comparison, denotes the greater larvicidal effectiveness of the nanoemulsion.

Volpato et al. [38], in 2016, evaluated the effect of *Cinnamomum zelanycum* EO on mealworm (*Alphitobius diaperinus*), an insect of Coleoptera order that causes poultry diseases by transmitting pathogenic bacteria and viruses. In the chemical analysis, the EO presented cinnamaldehyde as the major constituent. The nanoemulsions at a concentration of 5% caused 70% mortality of the larvae at the L8 stage after two days, and had a three-fold more pronounced effect when compared to treatment with unemulsified EO within 3 days. Moreover, the nanoemulsions showed no deleterious effects on survival and reproduction tests of springtails (*Folsomia candida*), evidencing that nanoencapsulation of cinnamon oil significantly reduced its toxic effects without altering the effectiveness in controlling *A. diaperinus*.

More recently, in 2017, larvicidal activity against *A. aegypti* was also demonstrated by the work of Botas et al. [39]. They produced nanoemulsions based on EO of *Baccharis reticulata*, as well as D-limonene, its major component (25%). The two nanoemulsions caused mortality of the larvae of *A. aegypti*, with LC_50_ values of 118.94 µg/mL and 81.19 µg/mL, respectively. These results reinforce the importance of D-limonene as the main active agent of the EO composition, probably by inhibition of acetylcholinesterase, an activity also demonstrated through this study. In the same year, Balasubramani et al. [40] described a similar effect on *A. aegypti* larvae. In this work, the nanoemulsion of leaf EO from *Vitex negundo* L. was active against 2nd and 3rd instar larvae, after 12 and 24 h exposure periods. After a 12 h exposure period, the LC_50_ values of 2nd and 3rd instar larvae were 64.54 and 70.31 ppm, respectively. In comparison, the values of the non-emulsioned EO were 118.15 and 92.63, respectively. Likewise, after a 24 h exposure period, the nanoemulsion LC_50_ values were 28.84 and 43.29 ppm, whereas the non-emulsioned EO values were 77.35 and 56.13 ppm, respectively.

Still more recently, researchers from Iran published a work about inhibition of *Anopheles stephensi*, a major vector of malaria in their country, by the action of the nanoemulsion-based in EO from *Artemisia dracunculus* (Asteraceae). A bioassay of nanoemulsion on 3rd and 4th instar larvae was performed, with LC_50_ or LC_90_ of 11.36 or 17.54 ppm, respectively. The major EO constituent was *p*-allylanisole [41]. Similarly, one year later, some of these authors also evaluated the larvicidal effect on *A. stephensi* by the action of nanoemulsionated EO from *Anethum graveolens* (Umbelliferae), which presented *p*-cymene and α-phellandrene as main compounds. After 1 h of contact, larvicidal activities of 50 and 90% were found at 38.8 and 65 ppm, respectively, against 3rd and 4th instar larvae of *A. stephensi*. The authors considered it to be a preparation with appropriate activity against larvae of *A. stephensi* with no minimum environmental effect [42]. Moreover, in comparison with a similar study with *A. graveolens* non-emulsified EO, the nanoemulsion showed more effectiveness, since the oil LD_50_ without nanoemulsification was 100 ppm after the same contact time [43].

In late 2018, Sundararajan et al. [44] showed the activity of the nanoemulsionated leaf EO from *Ocimum basilicum* on 2nd and 3rd instar larvae of *C. quinquefasciatus*. *Trans*-β-Guaiene (16.89%) and α-Cadinol (15.66%) were the major compounds of this EO. The larvicidal activity was observed after a 24 h exposure period, and significant mortality was observed after the treatments with nanoemulsion, in which the 2nd and 3rd instar larvae exhibited maximum mortality at 100 ppm (96.87 ± 0.55% and 93.89 ± 0.55%, respectively). All studies described in this section are shown in Table 1.

### 2.2. Insecticidal and Repellent Activity

Other scientific groups also have conducted studies on adult insects, as well as on other insect development stages. In 2017, Ramar et al. [45] evaluated the effect of different nanoformulations of EO from *Ocimum sanctum* on *A. aegypti* and *C. quinquefasciatus* adult species. Through the filter paper impregnation method, the formulation containing 30% of EO caused 98% (*A. aegypti*) and 100% (*C. quinquefasciatus*) of knock down activity when diluted to a concentration of 50 mg/cm^2^.

In 2008, Sakulku et al. [46] showed the repellent effect of a citronella EO nanoemulsion against *A. aegypti*, using the human-bait technique, based on standard test of World Health Organization (WHO). The nanoemulsion containing 20% oil and 1:1 glycerol-water ratio resulted in a high protection time (2.8 h), which can be considered high in comparison with EO diluted to 10% in olive oil, which, in turn, showed a protection time of only 54.75 min. D-limonene and citronellal were the principal substances of the EO (40% each) and have repellent activity [47].

One year later, Nuchuchua et al. [48] reported the repellent effect of nanoemulsions from different EOs on *A. aegypti* adults. In this study, seven formulations were made using different proportions of oils from *Cymbopogon nardus* (citronella oil), *Ocimum americanum* (hairy basil) and *Vetiveria zizanioides* (vetiver). Using the human-bait technique, the formulation with percentage concentration of 10:5:5 (citronella, hairy basil and vetiver) produced a protection time of 4.7 h. Limonene and citronellal (citronella), 3-carene and caryophyllene (hairy basil) and vetiveric acid (vetiver) were the major compounds in the EOs, and probably acted through synergic mechanisms. All nanoemulsions described in this section are shown in Table 2.

### 2.3. Acaricidal Activity

*Rhipicephalus microplus* is an Ixodidae tick that causes severe economic losses on livestock, and it is the main vector of tick-transmitted agents such as *Babesia bigemina*, *B. bovis* and *Anaplasma marginale*. Some groups of researchers have reported the acaricidal activity of EO nanoemulsions against this species. Santos et al. [49] demonstrated the activity of the nanoemulsion-based in 5% *Cinnamomum verum* EO that caused 97% and 63.5% of oviposition inhibition, in vitro and in vivo, respectively. In comparison, other study made by Monteiro et al. [51] showed that the non-emulsioned EO did not caused oviposition inhibition. Moreover, after 20 days, the cows that were previously infested and treated with the nanoemulsion were free of the parasites [49]. Similar work done by Galli et al. [50] and contributors with *Eucalyptus globulus* nanoemulsion showed that the formulations at 1% and 5% inhibited parasite reproduction by 50% and 61.2%, respectively. All oil nanoemulsions with acaricidal activity are shown in Table 2.

### 2.4. Antiparasitic Activity on Etiological Agents

*Trypanosoma evansi* is a flagellate parasite and the etiological agent of the disease known as “Surra” and “Mal das Cadeiras” in Brazilian horses and rarely affects humans. In 2013, Baldissera et al. [52] evaluated the in vitro trypanocidal activity of the nanoemulsified *Schinus molle* EO. The nanoemulsion at 0.5% and 1% were able to reduce the number of living parasites in 81% and 100%, respectively. When these results were compared with the non-emulsified EO, this one showed a lower mortality rate, with 63% and 68%, respectively. In 2017, Ziaei et al. [53] observed the in vitro effect of *Lavandula officinalis* EO nanoemulsion on *Trichomonas vaginalis*, a flagellated parasite that causes trichomoniasis. At the concentration of 100 µg/mL, the nanoemulsion showed 81.9% of growth inhibition and exhibited low toxicity and macrophages (90.9% viability).

In 2017, Shokri et al. [54] also investigated the antiparasitic activity of nanoemulsionated EO of a *Lavanudula* species. The nanoemulsion of *L. angustifolia*, as well as of *R. officinalis*, showed an antileishmanial effect against *Leishmania major*, one of the etiological agents of cutaneous leishmaniasis in Iran. 1,8-cineol (22.29%) and linalool (11.22%) were the major compounds of *L. angustifolia* EO. The nanoemulsions of *L. angustifolia* and *R. officinalis* EOs showed antileishmania activity on promastigote with IC_50_ = 0.11 μL/mL and IC_50_ = 0.08 μL/mL, respectively. Compared to a similar study with the non-emulsioned EO of *R. officinalis*, this one showed IC_50_ = 2.6 μL/mL [55], which demonstrates a greater potency of the nanoemulsion to cause the mortality of the parasite. During the amastigote assay, both nanoemulsions were effective at least in concentration of 0.12 μL/mL and 0.06 µL/mL respectively, on mean infected macrophages (MIR) and amastigotes in macrophages [54]. In the same year, Moazeni et al. [56] evaluated the effect of *Zataria multiflora* EO on *Echinococcus granulosus sensu lato*, which causes cystic echinococcosis, a zoonotic infection with economic and public health importance in worldwide. The effect was observed on the protoscoleces form, originated in liver hydatid cysts collected from naturally infected sheep. The in vivo results showed that the scolicidal power of the nanoemulsion at concentration of 1 mg/mL was 88.01% and 100% after 10 and 20 min, respectively, while at a concentration of 2 mg/mL, the formulation showed 100% of scolicidal power after 10 min. When compared with a study made by Mahmoudvand et al. [57] in 2017, with non-emulsified oil, the nanoemulsion showed a more pronounced effect, since the EO alone at a concentration of 3125 μL/mL caused only 43.3% and 78.6% mortality after 10 and 20 min of exposure time, respectively. The in vivo studies revealed that the sizes of the largest cysts, as well as the total number of the cysts, were significantly lower in the mice treated with nanoemulsion [56]. It has been previously reported that thymol is the main compound of *Z. multiflora* EO [58] and is involved with the scolicidal activity [57,59,60] and destructive effect on the germinal layer of hydatid cysts [61]. All studies described in this section are shown in Table 3.

In general, recent works on the use of nanoemulsions with EOs as active agents in the control of diseases caused by different agents have reported the diversity of targets and efficiency when compared to other agents already used, as well as their simplicity of production [62,63]. The technology of nanoemulsion production provides several advantages with respect to the dispersed active principle and formulation stability, such as (i) better protection of the active against chemical or biological degradation, (ii) lower probability of creaming or sedimentation of droplets, (iii) greater contact surface of the target with the droplets that contains the active agent, (iv) possibility of dispersion of immiscible substances in a certain solvent, which in the case of EOs is usually water, besides the simplicity of production, (v) low cost of reagents, and (vi) less residual damage to the environment when compared to synthetic products, widely used in modern times [22,27,63].

Regarding the potentialization of the action of the dispersed active in the formulation, this is the main advantage provided by the nanoformulations, since the EOs have serious problems of dispersion in aqueous vehicles and that mainly, in the case of contact with insects and other vectors, who have a large part of their cycle or living environment in contact with water, this is a limiting factor for the effectiveness of the action [24,25]. These facts can be observed when some results showed in the present review are compared with similar studies made with non-emulsified oils in which the EOs alone were not able to equalize the activity extension of the nanoformulations.

The diversity of the constituents of the EOs, mainly monoterpenes, sesquiterpenes and phenylpropanoids, contributes to the variety of mechanisms of action in the control of vectors and etiological agents of infectious/parasitic diseases, increasing the possibility of synergistic effect [64,65,66]. Some studies have described the probable toxicological or pharmacological effects of nanoemulsified EOs, such as eucalyptus oil, which decreased protein levels and caused inhibition of enzymes such as acetylcholinesterase and acid phosphatase in *C. quinquefasciatus* larvae [31], as well as the probably anticholinesterasic effect of *B. reticulata* EO on *A. aegypti* larvae [32]. In both cases, terpenes were the major components of the EOs, whereas in other works that showed phenols and phenylpropanoids as major constituents, the mechanisms of actions presented other specificities, as for example in the works of Ghosh et al. [32] (methyl-chavicol) and Moazeni et al. [58] (thymol). Finally, the nanoemulsion process of EOs benefits the performance of the action of its active components by several physical, chemical and biological factors, making real the potential application of these vegetal metabolites in the control of infectious/parasitic diseases. 

## 3. Conclusions

The nanomodification of EOs, which are hydro-immiscible in nature, significantly improves life utility and effectiveness as a pesticide or antiparasitic. Nanoformulation requires a smaller quantity of EO to develop a formulation, and the use of bio-surfactants and water also makes these nanopesticides conducive and friendly to the environment. The removal of the volatile organic solvent from the pesticide formulation improves its bio-security property and makes it a “greener” strategy for the control of vectors of pathogenic diseases. A higher degree of delivery to the target of action, stability of formulation, water dispersion, low cost, and lower ecological toxicity make these essential oil nanoformulations very efficient and highly ecological. Based on the works incorporated in this study, it can be concluded that the nanopesticides can be an efficient tool with an eco-safe property and can be applied safely to the control of vectors of diseases in humans and animals. Moreover, other studies with EO nanoemulsions have also shown its antiparasitic activity on etiological agents, which considers the possible use of these formulations in the treatment of infections, as well as their prevention.

## Figures and Tables

**Table 1:** Larvicidal activity of essential oil nanoemulsions.

Specie	Common Name	Main Essential Oil Compound(s)	Emulsificant	Insect	References
*Anethum graveolens*	Dill	*p*--Cymene and α-phellandrene	Tween 20	*Anopheles stephensi*	[42]
*Artemisia dracunculus*	Tarragon	*p*-Allylanisole	Tween 20	*Anopheles stephensi*	[41]
*Baccharis reticularia*	Sand-Rosemary	D-limonene	Tween 80	*Aedes aegypti*	[39]
*Cinnamomum zeylanicum*	True cinnamon tree	Cinnamaldehyde	Tween 80	*Alphitobius diaperinus*	[38]
*Eucalyptus globulus*	Eucalyptus	1,8-cineole	Tween 80	*Culex quinquefasciatus*	[31]
*Ocimum basilicum*	Basil	Methyl-chavicol	Tween 20	*Aedes aegypti*	[32]
*Ocimum basilicum*	Basil	*trans*-β-Guaiene and α-Cadinol	Tween 80	*Culex quinquefasciatus*	[44]
*Rosmarinus officinalis*	Rosemary	1,8-cineole	Tween 20	*Aedes aegypti*	[34]
*Vitex negundo*	Chinese chaste tree	2(*R*)-acetoxymethyl-1,3,3-trimethyl-4t-(3-methyl-2-buten-1-yl)-1t-cyclohexanol	Tween 80	*Aedes aegypti*	[40]

**Table 2:** Insecticidal, repellent and acaricidal activity of essential oil nanoemulsions.

Specie	Common Name	Main Essential Oil Compound(s)	Emulsificant	Insect/Parasite	References
*Cinnamomum verum*	Cinnamomum	Cinnamaldehyde	Tween 80	*Rhipicephalus microplus* (Acaricidal activity)	[49]
*Cymbopogon nardus*	Citronella	D-limonene and citronellal	Montanov 82	*Aedes aegypti* (Repellent activity)	[46]
*Cymbopogon nardus, Ocimum americanum* and *Vetiveria zizanioides*	Citronella, Hairy Basil and Vetiver	D-limonene and citronellal (*C. nardus*), 3-carene and caryophyllene (*O. americanum*), vetiveric acid (*V. zizanioides*)	Montanov 82	*Aedes aegypti* (Repellent activity)	[48]
*Eucalyptus globulus*	Eucalyptus	1,8-cineole	Tween 80	*Rhipicephalus microplus* (Acaricidal activity)	[50]
*Ocimum sanctum*	Holy Basil	Not described	Not described	*Culex quinquefasciatus* and *Aedes aegypti* (Inseticide activity)	[45]

**Table 3:** Antiparasitic activity of essential oil nanoemulsions.

Species	Common Name	Main Essential Oil Compound(s)	Emulsificant	Parasite	References
*Lavandula angustifolia* and *Rosmarinus officinalis*	Lavander and Rosemary	1,8-cineole and linalool	Tween 80	*Leishmania major*	[54]
*Lavandula officinalis*	Lavender	1,8-cineole	Tween 80	*Trichomonas vaginalis*	[53]
*Schinus molle*	Peruvian peppertree	Not Described	Tween 20	*Trypanosoma evansi*	[52]
*Zataria multiflora*	Avishan Shirazi	Thymol	Tween 80	Protoscoleces of the hydatid cysts	[56]
